# Tracheobronchial aspiration affects the outcome of hospitalization among Hepatic Encephalopathy patients

**DOI:** 10.12669/pjms.38.4.5114

**Published:** 2022

**Authors:** Qamar Rafiq, Mubashar Zeeshan, Ghulam Mustafa, Muhammad Irfan

**Affiliations:** 1 Qamar Rafiq, FCPS, GMC/Teaching Hospital, Gujranwala, Pakistan; 2 Mubashar Zeeshan, FCPS-I, Liver Clinic, Jail Road, Lahore, Pakistan; 3 Ghulam Mustafa, Ph.D, Department of Computer Sciences, Bahria University, Lahore, Pakistan; 4 Muhammad Irfan, FCPS, GMC/Teaching Hospital, Gujranwala, Pakistan

**Keywords:** Liver Cirrhosis, Complications, Hepatic Encephalopathy, Tracheobronchial Aspiration, Tertiary Care Hospital

## Abstract

**Objectives:**

The present study aimed to determine the effect of tracheobronchial aspiration on hospitalization outcomes and the factors influencing its occurrence.

**Methods:**

This prospective descriptive study was conducted from January 2017 to December 2020 at GMC/DHQ-Teaching Hospital in Gujranwala, Pakistan. All consenting liver cirrhosis patients with hepatic encephalopathy > 12 years of age admitted at the study site were included. The patient’s baseline characteristics and the hospitalization outcomes were noted in terms of death and discharge. The collected data was analyzed using SPSS version 22.0. The predictors of tracheobronchial aspiration were determined using Independent Sample T test and Chi-square test for quantitative and qualitative variables respectively. The p-values were taken statistically significant if < 0.05. A binary logistic regression analysis was performed to ascertain the effect of significant factors on the likelihood of tracheobronchial aspiration.

**Results:**

Among the total of 294 patients, 28.0% died during hospitalization. Death occurred significantly more in group of patients who had tracheobronchial aspiration (62.7% vs 12.0%, p<0.01). Increasing age was associated with increased chance of tracheobronchial aspiration (p<0.01). Male gender (49.6% vs 8.2%, p<0.01), patients whose hepatic encephalopathy was precipitated by upper GI bleed (59.7% vs 22.9%, p<0.01), and patients with comorbidities (p=0.02) were significantly prone to tracheobronchial aspiration.

**Conclusions:**

Tracheobronchial aspiration is a significant predictor of inpatient mortality among patients with hepatic encephalopathy. Male gender, increasing age & upper GI bleed predict aspiration in hepatic encephalopathy patients.

## INTRODUCTION

Liver cirrhosis is suggested to be one of the significant public health concerns in Pakistan.^1^ The most common etiology of liver cirrhosis in Pakistan is hepatitis C virus (HCV) infections, with estimated prevalence, ranges from 4.5% to 9.5% globally.^2^,^3^ However, the exact prevalence of this disorder worldwide is still unknown.^4^ Cirrhosis is usually an advanced form of progressive hepatic fibrosis that mainly occurs due to distortion of the hepatic architecture and the regenerative nodule.^4^,^5^ Chronic viral hepatitis, non-alcoholic fatty liver disease and alcohol consumption are suggested to be the most common cause of liver cirrhosis.^1^

Usually, the patient with this disease need to be hospitalized due to several complications, including upper gastrointestinal bleed, Spontaneous bacterial peritonitis (SBP), tense ascites, hepatopulmonary syndrome (HPS), Hepatocellular carcinoma (HCC), hepatic encephalopathy and Hepatic hydrothorax.^6^,^7^ It is seen that due to these complications, the hospital mortality rate of liver cirrhosis patients ranges from 13.5% to 35% worldwide, which is very high.^8^ Of these, hepatic encephalopathy is the major one; it features significant neuropsychiatric disturbances in response to liver dysfunction.^9^ The symptomatology varies, ranging from mild neurologic disclosures to overt coma.^10^ Furthermore, the disease fatality also precipitates due to tracheobronchial aspiration, which is primarily triggered in patients with neurologic trauma, motility disorders and those on a high dose of lactulose for the treatments of hepatic encephalopathy.^11^

Therefore, this study aimed to determine the effect of tracheobronchial aspiration on hospitalization outcomes and factors influencing its occurrence among hepatic encephalopathy patients presented to the tertiary care hospital of Gujranwala, Pakistan.

## METHODS

This prospective descriptive study was conducted at the Gujranwala Medical College / Teaching Hospital in Gujranwala, Pakistan, from January 2017 to December 2020. A total of 294 consenting hepatic encephalopathy patients > 12 years of age admitted at the study site were included. The baseline characteristics (age and gender) and the outcomes of the hospitalization in terms of death and discharge, were recoded. Tracheobronchial aspiration was established based on various parameters, i.e. wheeze and crackles along with tachypnea on chest examination, hypoxia on pulse oximetry (PO_2_ < 90 mmHg)^12^, or retrieving the food pieces or paste while suctioning of the airways, with or without specific opacities on posteroanterior x-ray imagings of the chest.^13^

The ethical review committee of Gujranwala Medical College approved the study protocol (Reference no 109/GMC; Dated 27 December 2016) and written informed consent were obtained from all the hospitalized patients or next of kin. The statistical analysis of the collected data was conducted on SPSS version 22.0. All the data variables included in this study were calculated as mean, percentage, standard deviation.

Independent sample T test and Chi-square test were used to compare different quantitative and qualitative variables respectively for the patients with or without tracheobronchial aspiration. The predictors of tracheobronchial aspiration were determined using odds ratios (OR), and 95% confidence intervals (CI) were estimated using logistic regression analysis, and p < 0.05 was considered significant.

## RESULTS

Out of 294 patients with hepatic encephalopathy, 28% died during hospitalization, while tracheobronchial aspiration was observed among 31.9% (94 out of 294). The age, weight, and CTP severity score of the patients suffering tracheobronchial aspiration were 63.88 ± 12.35 years, 73.45 ± 13.01Kg, and 10.56 ± 2.73 respectively. (Table-I).

**Table-I:** Associations of quantitative variables with Tracheobronchial aspiration in patients suffering hepatic encephalopathy (n = 294)*.

Quantitative variables	Tracheobronchial aspiration		Mean difference	p-value

	Yes (mean + SD)	No (mean + SD)		
1. Age (years)	63.88 + 12.35	52.48 + 13.19	11.41	<0.01
2. Weight (Kilogram)	73.45 + 13.01	72.52 + 13.09	0.93	0.571
3. CTP severity score	10.56 + 2.73	9.50 + 2.93	1.07	<0.01

* Independent sample T-test was used

Death occurred significantly more in group of patients who had tracheobronchial aspiration (62.7% vs 12.0%, p<0.01). Among factors that influenced tracheobronchial aspiration, male gender was more prone to tracheobronchial aspiration. 49.6% male (64 out of 129) & only 18.2% female (30 out of 165) suffered tracheobronchial aspiration (p<0.01). Similarly, tracheobronchial aspiration was significantly common among patients whose hepatic encephalopathy was precipitated by upper GI bleed (UGIB). 59.7% patients with UGIB and 22.9% without UGIB suffered tracheobronchial aspiration (p<0.01). Increasing age was associated with increased chance of tracheobronchial aspiration (p<0.01). The presence of comorbidities was also the predictor of tracheobronchial aspiration (p=0.02). However, tracheobronchial aspiration had no statistical association with hepatorenal syndrome (p=0.187) & space occupying hepatic lesion on imaging (p=0.594) among patients who presented with hepatic encephalopathy (Table-II).

**Table-II:** Associations of qualitative variables with Tracheobronchial aspiration in patients suffering hepatic encephalopathy (n = 294)*.

Predictors / Factors	Tracheobronchial aspiration		Total (n=294)	p-value

	Yes (n=94)	No (n=200)		
***Gender:***				
Male	64 (68.1%)	65 (32.5%)	129 (43.9%)	<0.01
Female	30 (31.9%)	135 (67.5%)	165 (56.1%)	
***Outcome of hospitalization:***				
Death	59 (62.7%)	24 (12.0%)	83 (28.0%)	<0.01
No death	35 (37.2%)	176 (88.0%)	211 (72.0%)	
***Precipitating factor of Hepatic encephalopathy:***				
Upper GI bleed	43 (45.7%)	29 (14.5%)	72 (24.5%)	<0.01
Other than upper GI bleed	51 (54.3%)	171 (85.5%)	222 (75.5%)	
***Comorbidities:***				
Yes	22 (23.4%)	26 (13.0%)	48 (16.3%)	0.02
No	72 (76.6%)	174 (87.0%)	246 (83.7%)	
***Hepatorenal Syndrome:***				
Yes	20 (21.3%)	30 (15.0%)	50 (17.0%)	0.187
No	74 (78.7%)	170 (85.0%)	244 (83.0%)	
***Space occupying lesion liver on imaging:***				
Yes	15 (16.0%)	27 (13.5%)	42 (14.3%)	0.594
No	79 (84.0%)	173 (86.5%)	252 (85.7%)	

* Chi-square test for independence was used

A binary logistic regression analysis was performed to ascertain the effect of different qualitative and quantitative factors on the likelihood of tracheobronchial aspiration in patients suffering hepatic encephalopathy. The logistic regression model explained 62.4% (Nagelkerke R^2^) of the variance in the occurrence of complications and correctly classified 84.4.0% of cases. Increasing age of the patients was associated with an increased likelihood of tracheobronchial aspiration. Patients with female gender, and upper GI bleed as precipitating etiology of hepatic encephalopathy were significantly more likely to exhibit tracheobronchial aspiration among patients suffering hepatic encephalopathy. The presence of tracheobronchial aspiration in the patients admitted with hepatic encephalopathy had 15.36 higher odds of death than those who were not having any such complication (Table-III).

**Table-III:** Binary Logistic Regression Analysis to predict association of various factors with Tracheobronchial aspiration in patients suffering hepatic encephalopathy (n = 294)*.

Risk Factors	B	S.E.	Wald-Statistic	p-value	Odds Ratio	95% C.I. for EXP(B)	

						Lower	Upper
Gender (Male/Female)	2.191	.412	28.321	<0.01	8.946	3.991	20.048
Age (years)	-.087	.016	30.143	<0.01	.917	.889	.946
Weight (Kilogram)	-.003	.014	.044	0.834	.997	.971	1.024
CTP severity score	.009	.068	.018	0.893	1.009	.884	1.153
Outcome of hospitalization (Death/No death)	2.732	.431	40.075	<0.01	15.357	6.592	35.777
Precipitating factor of Hepatic encephalopathy (Upper GI bleed/Other)	2.156	.571	14.245	<0.01	8.638	2.819	26.465
Comorbidities (Yes/No)	.361	.714	.256	0.613	1.435	.354	5.818
Hepatorenal Syndrome (Yes/No)	-.685	.713	.923	0.337	.504	.125	2.039
Space occupying lesion liver on imaging (Yes/No)	-.755	.615	1.505	0.220	.470	.141	1.570
Constant	3.042	1.601	3.610	0.057	20.945		

Cox & Snell R Square = 44.6%, Nagelkerke R Square = 62.4%, Percentage correct =86.4%.

## DISCUSSION

The morbidity and mortality rates that are associated with the end of life in patients with liver cirrhosis and other associated complications are substantial.^14^ It is evident that a tracheobronchial aspiration is a common event in critically ill hepatic encephalopathy patients.^15^

The inpatient mortality rate associated with hepatic encephalopathy ranges between 14.13% to 15.61% worldwide, of which 30% is contributed by pulmonary aspiration.^16^ In the present study, the inpatient mortality rate of patients suffering from hepatic encephalopathy was 47.9%. We observed tracheobronchial aspiration in 31.97% of the total enrolled patients, while 84.0% of them died (Table-II). The hepatic encephalopathy patients with tracheobronchial aspiration were 15.89 times more likely to die as compared to the counterparts (p<0.01) (Table-III). In support, a similar study reports 12.392 times higher odds of dying among tracheobronchial aspiration patients than those who hadn’t experienced it^7^. Furthermore, a more recent study (2020) also suggested that tracheobronchial aspiration is a significant predictor of death among patients with hepatic encephalopathy [OR 10.150; (95% CI 4.13-22.14); p<0.01].^17^

Studies suggest that the presence of tracheobronchial aspiration in hepatic encephalopathy patients is multifactorial.^11^ According to a systematic review, male gender, increasing age, and PPI use significantly increase the risk of tracheobronchial aspiration.^18^ Similarly, in our study, the male gender (68.1%) was more prone to tracheobronchial aspiration (p<0.01), and it was prevalent among older adults (53.2%) (Table-II). In contrast, Manabe et al. conducted a univariate analysis and identified that older age was not a risk factor for tracheobronchial aspiration.^19^

Although the tracheobronchial aspiration carries high mortality risk, it is preventable.^20^ The tracheobronchial aspiration observed during upper gastrointestinal bleeding (UGIB) could be prevented through intubation^21^. Studies show that aspiration in patients with liver cirrhosis can be minimized during any endoscopic intervention by using safety measures.^22^,^23^

### Limitations of the Study:

The study demonstrated a high mortality rate primarily contributed by tracheobronchial aspiration, while no other reasons were studied which remains the major limitation of the present study. There may be other possible reasons than the ones studied (age, gender and occurrence of tracheobronchial aspiration) that need to be investigated. Furthermore, the present study did not include the findings of LFTs, RFTs, imaging study results, history of previous complications of cirrhosis, co-morbid issues.

## CONCLUSION

The results of our study suggests that tracheobronchial aspiration was a significant predictor of mortality among the patient suffering from hepatic encephalopathy in our studied population. The male gender and older patients displayed a higher frequency of tracheobronchial aspiration during hospitalization than the counterparts. Furthermore, hepatic encephalopathy patients whose precipitating factor was upper GI bleed suffered more tracheobronchial aspiration as compared to the patients in which etiology of hepatic encephalopathy was other than upper GI bleed.

### Authors’ Contribution:

**QR:** Substantial contributions to conception & design, acquisition of data, analysis & interpretation of data.

**MZ:** Drafting the article and revising it critically for important intellectual content.

**GM:** Final approval of the version to be published.

**MI:** Agreement to be accountable for all aspects of the work in ensuring that questions related to the accuracy or integrity of any part of the work are appropriately investigated and resolved.
